# Chitosan-based strategies as eco-friendly solutions for controlling *Brettanomyces bruxellensis* contamination in wine production

**DOI:** 10.3389/fmicb.2025.1631987

**Published:** 2025-07-15

**Authors:** Francesco Tedesco, Antonino Biundo, Antonio Caporusso, Isabella Pisano, Gabriella Siesto, Rocchina Pietrafesa, Chloé Abry, Alexandre Hervé, Patrizia Falabella, Angela Capece

**Affiliations:** ^1^Università del Salento, Dipartimento di Scienze e Tecnologie Biologiche ed Ambientali, Lecce, Italy; ^2^Spin-off StarFInn s.r.l.s., Dipartimento di Scienze Agrarie, Forestali, Alimentari ed Ambientali, Università degli Studi della Basilicata, Potenza, Italy; ^3^REWOW srl, Bari, Italy; ^4^Italian National Agency for New Technologies, Energy and Sustainable Economic Development (ENEA), Energy Technologies and Renewable Sources Department, ENEA, Rotondella, Italy; ^5^Department of Biosciences, Biotechnology and Environment, University of Bari Aldo Moro, Bari, Italy; ^6^Interuniversity Consortium for Biotechnology (CIB), Trieste, Italy; ^7^Dipartimento di Scienze Agrarie, Forestali, Alimentari ed Ambientali, Università degli Studi della Basilicata, Potenza, Italy; ^8^Université Bourgogne Europe, L'Institut Agro, INRAE, UMR PAM, Dijon, France; ^9^Dipartimento di Scienze di Base e Applicate, Università degli Studi della Basilicata, Potenza, Italy; ^10^Spin-Off XFlies s.r.l., Università degli Studi della Basilicata, Potenza, Italy

**Keywords:** chitosan, *Brettanomyces bruxellensis*, wine spoilage, antimicrobial activity, insect-derived products, sustainable biocontrol

## Abstract

**Introduction:**

This study investigates the antimicrobial activity of chitosan against *Brettanomyces bruxellensis*, a wine spoilage yeast responsible for producing volatile phenols that lead to undesirable sensory defects commonly referred to as “Brett” character. The most widely used antimicrobial compound in oenology is sulphur dioxide (SO_2_), due to its broad spectrum of action, but growing consumer demand for reduced chemical additives and evidence of *Brettanomyces* spp. resistance to it, have encouraged different alternative strategies. Among these, chitosan has been accepted for the control of *Brettanomyces* yeasts.

**Methods:**

In this study, some *B. bruxellensis* strains were treated with different types of chitosan: a commercial product (chitosan extracted from shrimp shells), a fungal origin chitosan approved for oenological use and an insect-derived chitosan, which is used for the first time in oenology as *Brettanomyces* control strategy. The effects on yeast cells were assessed through analysis of cell wall composition, flow cytometry to evaluate cell viability and membrane integrity, and optical and electronic microscopic observation.

**Results and discussion:**

Our results indicated that all chitosan types effectively reduced the yeast population, with commercial and insect-derived chitosan demonstrating higher efficacy than oenological one. These findings highlight insect-based chitosan as a promising, sustainable alternative for microbial control in wine production. Furthermore, its use supports circular economy principles, offering an eco-friendly solution reducing reliance on conventional chemical preservatives like SO_2_, contributing to support the development of new preservation methods with reduced environmental impact in the food industry.

## Graphical abstract

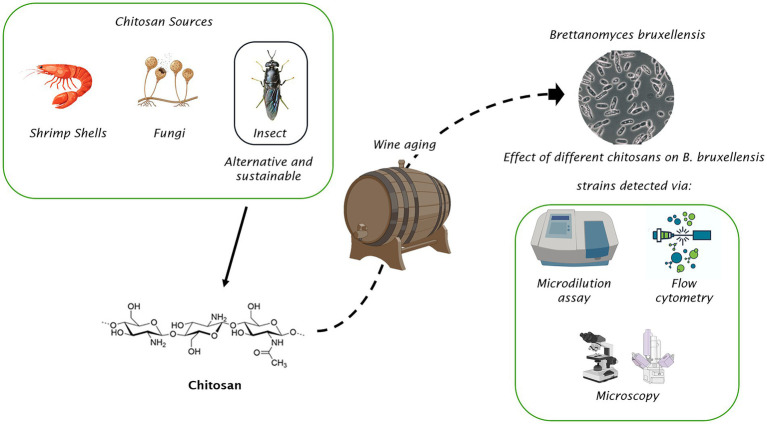

## 1 Introduction

*Brettanomyces bruxellensis* is widely recognized as a spoilage yeast in wine production due to its metabolic capability of converting hydroxycinnamic acids into volatile phenolic compounds, such as 4-ethylphenol and 4-ethylguaiacol, which significantly deteriorate the sensory properties of wine (Suárez et al., 2007; Kheir et al., 2013; Šućur et al., 2016). The detrimental impact of *B. bruxellensis* in wine is largely attributed to its ability to produce volatile phenols through enzymatic transformation. This process is primarily mediated by two key enzymes: cinnamate decarboxylase and vinylphenol reductase (Sturm et al., 2015; Lomascolo et al., 2023). Initially, cinnamate decarboxylase catalyzes the conversion of hydroxycinnamic acids, such as *p*-coumaric acid and ferulic acid, into intermediates like 4-vinylphenol and 4-vinylguaiacol, respectively (Van beneden et al., 2008). These intermediates are then reduced by vinylphenol reductase to 4-ethylphenol and 4-ethylguaiacol, respectively, (Milheiro et al., 2019), which impart off-flavors characteristics (Perestrelo et al., 2019). The sensory threshold for 4-ethylphenol is approximately 230 μg/L, while for 4-ethylguaiacol, it is around 47 μg/L (Csikor et al., 2018). When these thresholds are exceeded, the wine aroma and overall quality are negatively affected, with off-flavors characteristics, such as “horse sweat,” “stable,” and “leather” (Perestrelo et al., 2019), leading to severe economic losses for the wine industry.

*Brettanomyces* spp. exhibits remarkable adaptability to harsh environmental conditions that are typically unfavorable to other microorganisms. As a facultative anaerobe, this yeast species can thrive in both oxygen-rich and oxygen-deprived environments (Ciani and Ferraro, 1997; Aguilar Uscanga et al., 2003). Furthermore, *B. bruxellensis* demonstrates high resistance to ethanol, low pH levels, and osmotic stress, making it particularly challenging to eradicate from winery environments (Smith and Divol, 2016). One of the most critical points of contamination occurs during the wine aging process, particularly in wooden barrels. In this environment, the porous nature of wood promotes yeast penetration and biofilm development, providing a reservoir for persistent contamination (Cartwright and Edwards, 2020). Additionally, oxygen uptake during barrel aging can create an environment conducive to *Brettanomyces* proliferation, aggravating the risk of spoilage (Rubio et al., 2015).

Early detection of *B. bruxellensis* is crucial for implementing effective control measures and preventing large-scale contamination.

Sulfur dioxide (SO_2_) remains the most employed antimicrobial agent in winemaking due to its broad-spectrum activity against microorganisms, including *B. bruxellensis* (Dimopoulou et al., 2019). However, studies have demonstrated variability in strain-specific resistance to SO_2_, with some strains capable of surviving even at molecular SO_2_ concentrations of 0.6 mg/L (Vigentini et al., 2013). Additionally, exposure to SO_2_ can induce a viable but non-culturable (VBNC) state in *B. bruxellensis*, allowing the yeast to persist in the wine and potentially reactivate under favorable conditions (Agnolucci et al., 2010; Zuehlke and Edwards, 2013). The persistence of contamination presents with significant challenges for effective control, thereby needing the exploration of alternative antimicrobial approaches (Tedesco et al., 2022). Additionally, the overuse of sulfites poses potential health risks to consumers, given their association with allergic responses and other negative health outcomes (Lester, 1995; Vally and Thompson, 2001), prompting to impose strict limits on their use in food and beverage products.

In recent years, studies about alternative antimicrobial compounds have been focused on natural materials, such as chitosan and chitin (Fernández-Pan et al., 2015; Shekarforoush et al., 2015). Particularly, chitosan has emerged as a promising alternative for controlling *B. bruxellensis* contamination in wine (Petrova et al., 2016; Paulin et al., 2020). Chitosan is a biopolymer derived from chitin, the second most abundant polysaccharide in nature after cellulose; it has been proved to be non-toxic, biodegradable and biocompatible (El-Araby et al., 2024). Chitosan is approved as Generally Recognized as Safe (GRAS) by the U.S. Food Drug Administration (FDA) and it has a broad-spectrum of antimicrobial activity against both Gram-positive and Gram-negative bacteria as well as fungi (Harish Prashanth and Tharanathan, 2007; Ke et al., 2021b). Antimicrobial activity of chitosan depends on different factors. Indeed, the effectiveness of the antimicrobial activity of chitosan is highly affected on the target microorganism (Kong et al., 2010; Hosseinnejad and Jafari, 2016; Varlamov and Mysyakina, 2018), combined with intrinsic features of chitosan, such as the molecular weight and the deacetylation degree, and the pH of the medium (Zheng and Zhu, 2003; Goy et al., 2009; Kong et al., 2010). Furthermore, the activity against microorganisms can be classified as extracellular, intracellular, or both on the basis of the targeting site of antimicrobial actions (Kong et al., 2010; Varlamov and Mysyakina, 2018). The high-molecular weight chitosan, which is unable to cross the target microorganisms cell wall and membrane, has shown the potential antimicrobial activities involving a chelation activity on essential metals, preventing nutrient uptake and modifying cell permeability (Rabea et al., 2003; Kravanja et al., 2019). The low-molecular weight chitosan, in addition to these extracellular activities, has also intracellular actions, addressed toward RNA, protein synthesis, and mitochondrial function (Ke et al., 2021a).

The International Organization of Vine and Wine (OIV) (2009) has approved the use of chitosan extracted from the fungus *Aspergillus niger* for antimicrobial applications in winemaking. Studies indicate that chitosan application can help manage contamination levels without adversely affecting wine composition, offering an innovative solution to this industry challenge (Zuehlke and Edwards, 2013).

While fungal chitosan is currently approved for oenological use, alternative sources such as crustaceans and insects, are gaining attention due to their sustainability and potential functional advantages (Tedesco et al., 2024). Crustacean-derived chitosan is not permitted in winemaking, primarily due to concerns over allergenicity and potential contamination with fish proteins (Amaral et al., 2016; Rocha et al., 2017). In contrast, insect-derived chitosan represents a promising alternative, as insects are not classified as major allergens by the U.S. FDA (de Gier and Verhoeckx, 2018). Insect-derived chitosan offers additional benefits, including year-round availability (Nakamura et al., 2016), and the potential for sustainable bioconversion of organic waste (Salomone et al., 2017; Fadhillah and Bagastyo, 2020). Among insect species, the black soldier fly (*Hermetia illucens*) has been identified as a promising source of chitin (Triunfo et al., 2024). Chitosan extracted from this species is characterized by a low molecular weight (MW) and a high deacetylation degree (DD) (Triunfo et al., 2022); its chemical–physical characteristics can affect some properties, such as its antimicrobial activity (Omura et al., 2003), that is particularly useful in some fields of application, such as the food industry (Tafi et al., 2023; Triunfo et al., 2023a, 2023b; Guarnieri et al., 2024). Recent developments, in fact, are focusing attention on using chitosan to create natural edible protective films. For example, the creation of microstructures by adding chitosan to synthetic melanin-like nanoparticles (MNP) or D-*α*-tocopheryl polyethylene glycol 1,000 succinate (TPGS) or silicon dioxide nanoparticles (nano-SiO_2_), would allow the creation of intelligent films with improved properties for active food packaging, increasing protection from moisture and oxygen (Bi et al., 2020; Roy et al., 2020). Other studies, however, have demonstrated the possibility of grafting an antifungal agent, extracted from fermented lemon peel, to chitosan to create a film to protect citrus fruits in the post-harvest period (Arslan et al., 2024). Furthermore, Sultan et al. (2021) created a coating film based on chitosan, beeswax and pollen grains to improve physical and mechanical properties. Finally, recent studies have also demonstrated the applicability of chitosan extracted from insects as an alternative to chitosan extracted from crustaceans for the preservation of fresh fruit (Guarnieri et al., 2024).

The aims of the study were: (i) to compare the antimicrobial activity against *B. bruxellensis* of chitosan from different sources, such as insects (an alternative and sustainable source), shrimp shells and oenological chitosan (extracted from fungi); (ii) to evaluate the effects of these chitosans on yeast cell viability and membrane integrity by using different analytical techniques, including flow cytometry, optical and electron microscopy, and biochemical assays; (iii) to contribute to the development of sustainable solutions for the control of *B. bruxellensis* contamination in wine, addressing the ongoing challenges associated with *Brettanomyces* spoilage.

## 2 Materials and methods

### 2.1 Yeast strains and chitosan polymers

Five strains of *Brettanomyces bruxellensis* and one *Saccharomyces cerevisiae* strain were included in this study. The *B. bruxellensis* strains were: LO417, isolated in Bordeaux from red wine by the Institut des Sciences de la Vigne et du Vin (ISVV, Bordeaux, France); LO2e2, isolated by the Institut Technique de la Vigne et du Vin (Beaune, France) (Lebleux et al., 2021); CBS 4601, CBS 4481, CBS 2499 (*Brettanomyces*/*Dekkera bruxellensis*), belonging to CBS-KNAW culture collection. As regards the *S. cerevisiae* strain, 4LBI-3 was indigenous yeast, isolated during spontaneous grape must fermentations and belonging to UNIBAS Yeast Collection (UBYC) (University of Basilicata Potenza, Italy) (Caruso et al., 2002; Peter et al., 2018).

The strains were routinely maintained at 4°C on YPD medium (2% glucose, 2% peptone, 1% yeast extract; Oxoid, Hampshire, United Kingdom) with 2% agar (Oxoid, Hampshire, United Kingdom) from stocks stored in cryogenic vials at −80°C.

Chitosan polymers from three sources were tested, which were shrimp shells (DD > 75%, MW = 190–375 kDa), purchased from Merck KGaA (Darmstadt, Hesse, Germany), insects (DD > 90%, MW = 80–100 kDa) extracted from *H. illucens* pupal exuviae and obtained from Xflies s.r.l (Potenza, Italy), following the methodologies described in Triunfo et al. (2022), and fungi (DD > 60%, MW = 400 kDa) extracted from *Aspergillus niger* and purchased from BioLaffort® (Bordeaux, France). For each one, stock solutions (10 g/L) were prepared, by solubilizing the chitosan in 1% (v/v) of glacial acetic acid 99% (Merck KGaA, Darmstadt, Hesse, Germany). The solutions were stirred overnight and sterilized by filtration (0.22 μm).

### 2.2 Sensitivity of *Brettanomyces bruxellensis* strains to chitosan from different sources

The study of sensitivity of the five *B. bruxellensis* strains (LO417, CBS 4481, CBS 2499, LO2e2, CBS 4601) to different chitosan types was carried out by microdilution assay. The test was performed in 96-well microtiter plates, containing Yeast Nitrogen Base (YNB) medium (Merck KGaA, Darmstadt, Hesse, Germany), added with increasing amounts of shrimp shells, insect-derived, fungal chitosan and acetic acid (the solvent used to solubilize the chitosan), following the protocol described by Guarnieri et al. (2022). A range of concentrations was evaluated, specifically 12.5, 25, 50, and 100 mg/L for the different chitosan types, and 0.0025, 0.005, 0.01, and 0.02% for acetic acid. YNB medium without antimicrobial agents was used as the control.

From a 48-h fresh pre-culture, the five *B. bruxellensis* strains were inoculated at concentration of 1 × 10^6^ cells/mL. The micro plates were incubated at 28°C and the growth was monitored measuring the optical density at 600 nm (OD_600_) after 24 h of incubation. All experiments were performed in triplicate and the strain resistance percentage to the different chitosan types was calculated as the ratio between the OD_600_ of the treated sample and the OD_600_ of the control.

### 2.3 Optical and electron microscopy

*Brettanomyces bruxellensis* cell samples treated with chitosan were observed both at optical and electron microscopy.

For optical microscopy, 1 mL of sample taken from the culture flasks was treated with a few drops of 0.1% (w/v) methylene blue (Merck KGaA, Darmstadt, Hesse, Germany); the damaged/dead cells appeared blue stained, while no staining occurs in live cells. The slides were observed under an optical microscope at 40x and 100x magnification and images were digitally captured.

For electron microscopy, a scanning electron microscope (Hitachi SU3800, SEM) was used. This SEM required a simple procedure for sample preparation: yeast cells from the culture flasks were loaded on metal stubs with carbon fiber, a conductive and stable material under the electron beam. The stubs were then placed in an incubator at 37°C to allow water evaporation and sample drying. After drying, the samples were coated with a thin layer of gold using a sputtering process to increase sample conductivity and enhance image quality. The stubs were finally placed on the sample stage in the sample chamber. The PC-SEM allowed for the observation and acquisition of images of the samples at various magnifications.

### 2.4 Flow cytometry analysis

The effects of the three chitosan types on yeast cells were evaluated using an Attune™ NxT Acoustic Focusing Cytometer (Thermo Fisher, Waltham MA, United States), by testing one *B. bruxellensis* strain (LO417), selected on the basis of previous results, and one *S. cerevisiae* strain (4LBI-3). Cells in the exponential growth phase were inoculated in 50 mL of Yeast Nitrogen Base (Merck KGaA, Darmstadt, Hesse, Germany) medium, at a concentration of 1 × 10^6^ cells/mL, and incubated at 28°C without stirring.

After 24 h of incubation, the yeasts has adapted to the new culture medium and were subjected to antimicrobial treatment with the addition of 100 mg/L of shrimp shells, insect-derived and fungal chitosan. As control, a sample without antimicrobial treatments was used.

Cell-counting and viability of the yeast cultures, after the chitosan treatments, were investigated over the time at 0, 2, 4, 6, 24, and 48 h of incubation.

At each sampling time, 3 mL of sample were collected and split into three sub-samples of 1 mL each (containing 1 × 10^6^ cells/mL). The collected cells were resuspended in Phosphate Buffer Solution (PBS) (Merck KGaA, Darmstadt, Hesse, Germany), and stained with fluorescent markers for specific cellular assessments: 100 μL/mL of Propidium Iodide (PI) to evaluate cell damage (Di Noia et al., 2024), 1 μL/mL of 7-Aminoactinomycin D (7-AAD) for viability assessments (Colacicco et al., 2022) and 23 μL/mL of 3,3′-dihexyloxacarbocyanine iodide (DiOC_6_(3)) to measure membrane potential (López et al., 2021).

PI-labeled cells were incubated in the dark for 20 min, 7-AAD-labeled cells were incubated on ice for 45 min, and DiOC_6_(3)-labeled cells were incubated in the dark for 10 min. For DiOC_6_(3) staining, after incubation, the samples were centrifuged at 1,800 rpm for 5 min, the supernatant was removed, and the cells were resuspended in 1 mL of deionized water before being immediately analyzed using Attune™ NxT 3.2 Software. Fluorescently labeled cells were detected using the BL3 channel for red fluorescence and BL1 channel for green fluorescence. PI-stained cells were considered as damaged, as this marker penetrates cells with compromised membranes. 7-AAD-stained cells were considered non-viable, as this DNA intercalating dye cannot enter cells with intact membranes. DiOC_6_(3) stained cells exhibiting fluorescence loss which were identified as having impaired membrane integrity, as this marker assesses mitochondrial membrane potential and, indirectly, cell viability.

### 2.5 Evaluation of negative cell surface charge

The negative charge of the cell wall of *B. bruxellensis* LO417 and *S. cerevisiae* 4LBI-3 was evaluated by using the Alcian blue assay. Alcian blue is a positively charged dye, and the assay measures the amount of dye absorbed by the yeast cells, which are negatively charged (Kordialik-Bogacka, 2011). The yeasts were inoculated into 20 mL of YPD medium, and the flasks were incubated in a shaking incubator at 26°C and 180 rpm.

After 24 h of incubation for *S. cerevisiae* strain and after 48 h for *B. bruxellensis* (times corresponding to exponential growth phases), OD_600_ of each yeast culture was measured to determine the cell concentration.

As reported by Kregiel et al. (2012), an amount of inoculated medium containing approximatively 5 × 10^7^ cells/mL was centrifuged at 4,000 rpm for 10 min. The supernatant was removed, and the cell pellets were resuspended in 1 mL of 0.02 M sodium acetate buffer (pH 4.0), for washing the cells.

The washed cell pellets were resuspended in 1.8 mL of Alcian blue dye (Merck KGaA, Darmstadt, Hesse, Germany) buffer solution (50 mg/L; 0.02 M sodium acetate buffer; pH 4.0) and the suspensions were incubated at 25°C for 30 min to allow the binding of Alcian blue to the yeast cell walls. After incubation, the samples were centrifuged again, and the supernatants were collected.

The optical density of the collected supernatants, which represent the not cell-bound solution, and the pure Alcian blue dye buffer (without yeast cells) were measured at 615 nm.

The results were expressed as percentage of Alcian blue retention (ABR %) by analyzed yeast cells.

### 2.6 **β**-glucan content detection

The β-glucan content in the cell walls of *B. bruxellensis* LO417 and *S. cerevisiae* 4LBI-3 strains was determined using an enzymatic kit provided by Megazyme (Wicklow, Ireland). The assay is based on the specific activity of enzymes that hydrolyze β-glucans into simple sugars, which can be quantified using colorimetric methods (McCleary and Draga, 2016). The kit involves an initial acid hydrolysis step followed by enzymatic digestion, where β-glucans are broken down into glucose, which is then quantified using a colorimetric reagent (Danielson et al., 2010).

The yeast strains were inoculated into flasks containing 20 mL of YPD culture medium. The flasks were incubated in a shaking incubator at 26°C and 180 rpm. After 24 h for *S. cerevisiae* and 48 h for *B. bruxellensis* of incubation, 10 mL of samples were centrifuged at 4700 rpm for 10 min to remove the supernatant and collect the cell pellet. Approximately 20 mg of the pellet was transferred into culture tubes and 0.4 mL of KOH (Merck KGaA, Darmstadt, Hesse, Germany) 2 M was added to each tube, which were placed on a magnetic stirrer for 30 min in an ice bath. After 30 min, 1.6 mL of 1.2 M sodium acetate buffer (pH 3.8) and 40 μL of Glucazyme™ (exo-1,3-β-glucanase, endo-1,3-β-glucanase, β-glucosidase and chitinase suspension) were added to each tube. The tubes were stirred on the magnetic stirrer in an ice bath for an additional 2 min and then incubated in a water bath at 40°C overnight without agitation.

After approximately 16 h, 10 mL of sterile deionized water was added to each tube, and the tubes were centrifuged at 3000 rpm for 10 min. Subsequently, 0.1 mL of the supernatant was transferred into two tubes for duplicate readings. Additionally, two reagent blank tubes, containing 0.1 mL of 200 mM sodium acetate buffer (pH 5.0), and two D-glucose standard tubes, containing 0.1 mL of D-glucose standard solution (1.5 mg/mL in 0.2% w/v benzoic acid), were prepared. An aliquot of 4 mL of glucose oxidase/peroxidase reagent (GOPOD) was added to all the tubes, followed by incubation in a water bath at 40°C for 20 min. After incubation, the absorbance of each solution was measured at 510 nm against the reagent blank.

The readings obtained were used to determine the beta-glucan content, using the Mega-Calc™ software provided by Megazyme (Wicklow, Ireland).

### 2.7 Statistical analysis

Statistical analysis of the entire dataset was performed using the program PAST (Hammer and Harper, 2001). One-way analysis of variance (ANOVA) followed by a *post hoc* comparison (Tukey’s HSD test) were carried out; *p* values < 0.05 were considered statistically significant.

## 3 Results

### 3.1 Sensitivity of *Brettanomyces bruxellensis* to three chitosan types

The sensitivity of five *B. bruxellensis* strains to chitosan from different sources, such as shrimp shells, insects and fungi, was studied by microdilution assay in 96-well microtiter plates.

**Figure 1:**
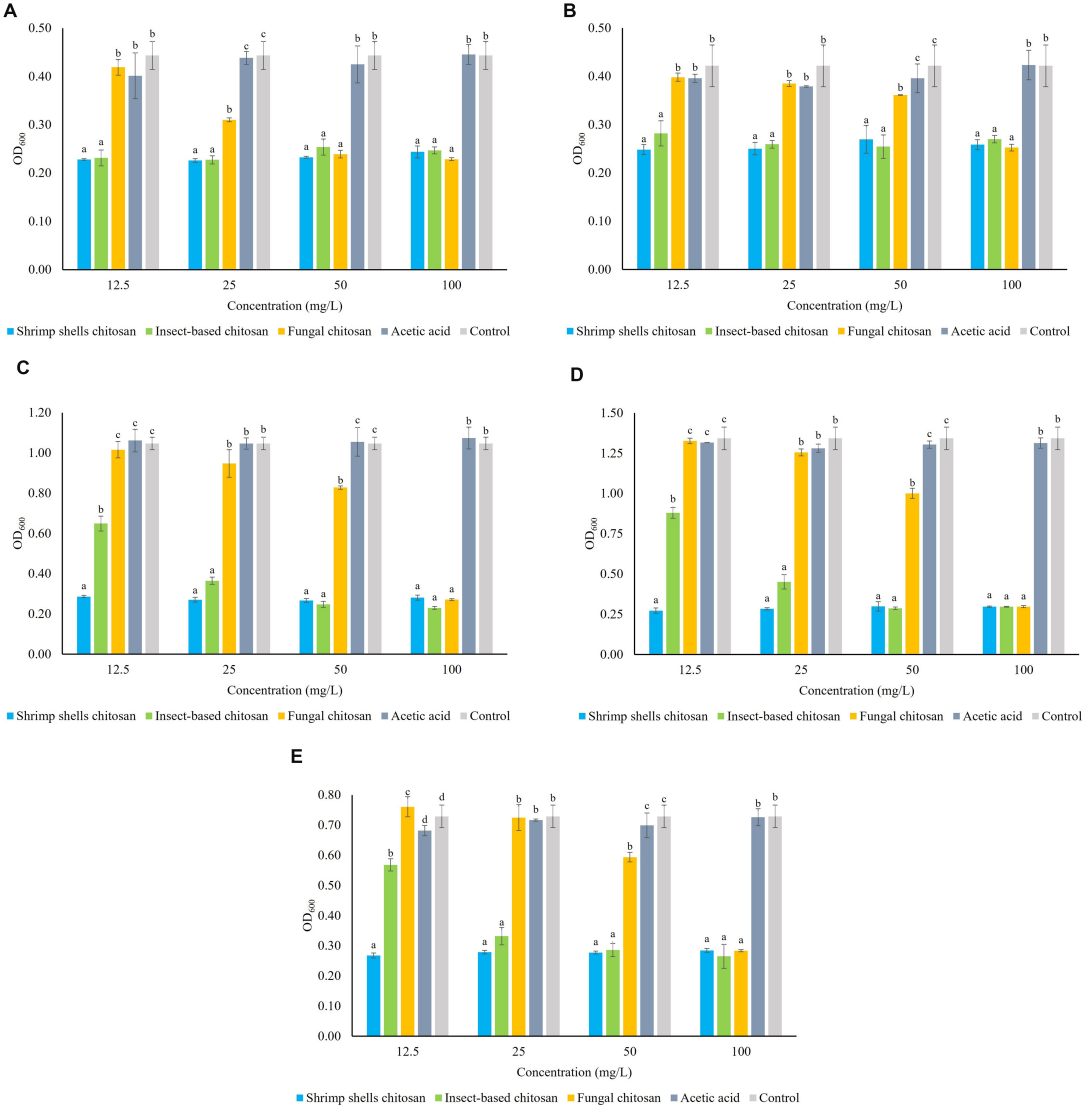
Sensitivity of five *Brettanomyces bruxellensis* strains LO417 **(A)**, CBS 4481 **(B)**, CBS 2499 **(C)**, LO2e2 **(D)**, CBS 4601 **(E)** to chitosan from different sources (shrimp shells, insects, fungi) at different concentrations (12.5, 25, 50, 100 mg/L). Acetic acid: medium added with amounts of acetic acid used as solvent for chitosan. Control: medium without chitosan. Data are the means of triplicate experiments ± standard deviation. Letters on plot bars indicate significant differences (*p* < 0.05) among various treatments.

Figures 1A–E show the microbial growth (OD_600_) of the five strains LO417, CBS 4481, CBS 2499, LO2e2, CBS 4601, 24 h after the treatment with the three chitosan types at increasing concentrations (12.5, 25, 50, 100 mg/L). As control, it was evaluated the growth level both in medium without chitosan and in medium containing acetic acid at the respective concentrations (0.0025, 0.005, 0.01, 0.02%), in order to exclude the antimicrobial activity of the solvent in which chitosan was solubilized. In all experimental conditions, acetic acid did not affect the growth of the microorganism, and the growth values were comparable to those observed in the control.

Chitosan derived from shrimp shells exhibits a strong antimicrobial effect across all the concentrations for all of *B. bruxellensis* strains.

The efficacy of fungal chitosan varies among the strains: for CBS 4481, CBS 2499, LO2e2 and CBS 4601 strains (Figures 1B–E), fungal chitosan begins to lose effectiveness at 50 mg/L and it has no effect on cell growth when the concentration is further reduced to 25 mg/L. Indeed, at this concentration the growth level of each strain is not statistically different from the growth in the controls. For LO417 strain (Figure 1A), fungal chitosan reduces its effectiveness at 25 mg/L and at 12.5 mg/L it loses its antimicrobial activity, with cell growth level similar to the controls.

On the other hand, the insect-derived chitosan, for strains LO417 and CBS 4481 (Figures 1A,B, respectively), it is effective as shrimp shells chitosan even at the lowest tested concentration of 12.5 mg/L. For strains CBS 2499, LO2e2, and CBS 4601 (Figures 1C–E, respectively), the antimicrobial effect of insect-derived chitosan begins to be shown at 12.5 mg/L, in which the cell growth level is lower than the controls, indicating antimicrobial activity.

**Figure 2:**
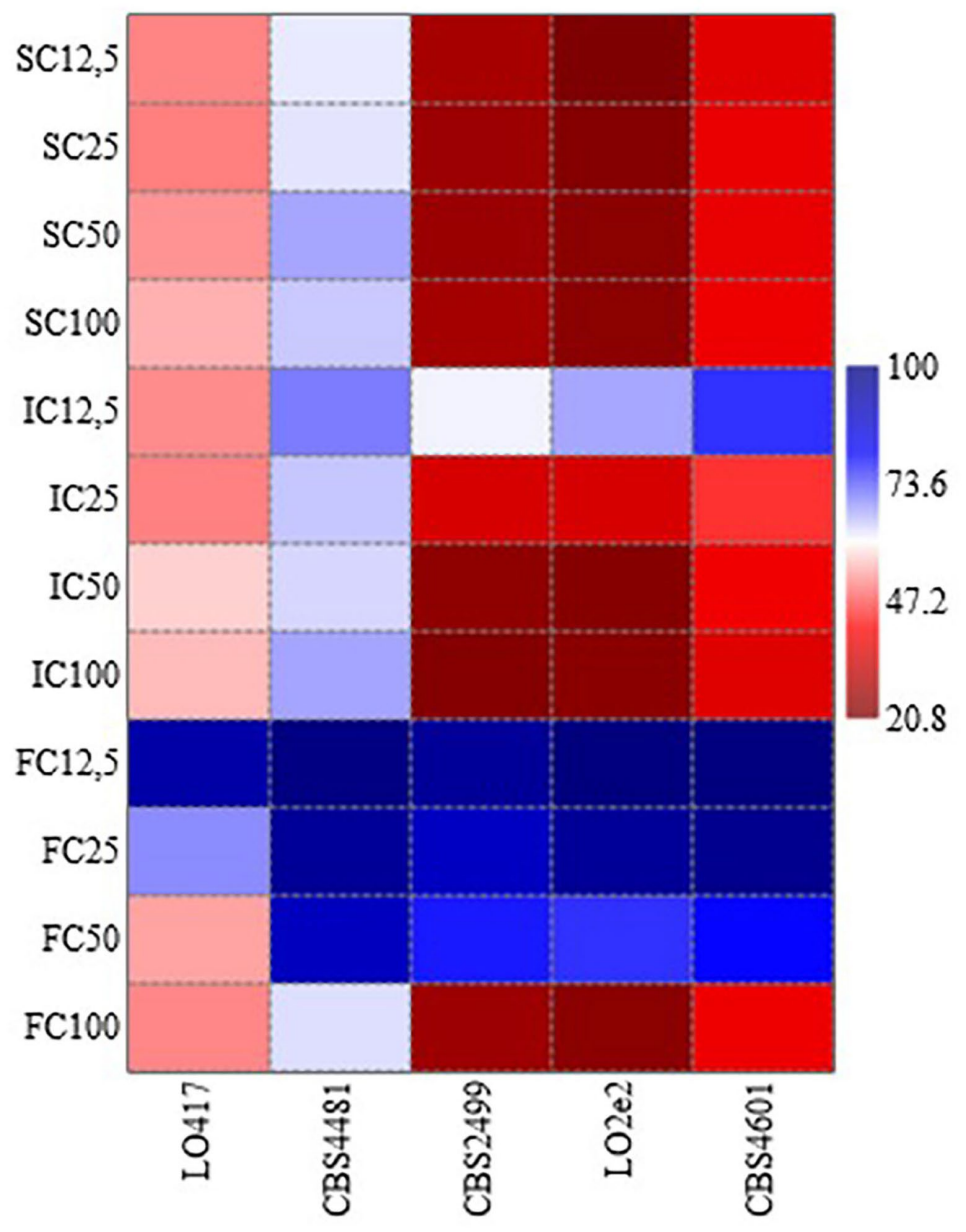
Heat map analysis summarizing the response of each *Brettanomyces bruxellensis* stain to chitosan type (SC, shrimp shells chitosan; IC, insect-derived chitosan, FC, fungal chitosan) and concentration (12.5, 25, 50 and 100 mg/L). Results were visualized using a false color scale, with red indicating lowest resistance level and blue the highest resistance level of each strain at each chitosan type and concentration.

Table 1 reports the resistance level of each strain toward the three chitosan types at 4 concentrations, calculated as ratio between level of growth of the treated samples and the control (measured as OD_600_). These data were elaborated by generating the heat map reported in Figure 2, in which the response of each *B. bruxellensis* stain to chitosan type and concentration is summarized.

**Table 1:** Resistance level of five *Brettanomyces bruxellensis* strains to chitosan from different sources at different concentrations.

*B. bruxellensis* strain code	Concentration (mg/L)	Shrimp shells chitosan (%)	Insect-derived chitosan (%)	Fungal chitosan (%)
LO417	12.5	50.95 ± 4.24^a^	51.51 ± 1.60^a^	93.71 ± 9.86^b^
	25	50.44 ± 3.03^a^	50.69 ± 1.93^a^	69.28 ± 6.01^b^
	50	51.96 ± 3.77^a^	56.78 ± 6.21^a^	53.32 ± 2.96^a^
	100	54.40 ± 4.43^a^	55.12 ± 3.25^a^	51.11 ± 4.52^a^
CBS 4481	12.5	62.03 ± 4.54^a^	70.51 ± 8.55^b^	99.46 ± 5.40^c^
	25	62.51 ± 4.52^a^	64.73 ± 3.10^a^	96.12 ± 4.78^b^
	50	67.32 ± 7.88^a^	63.52 ± 7.05^a^	90.18 ± 2.92^b^
	100	64.53 ± 4.66^a^	67.34 ± 0.38^a^	62.94 ± 0.95^a^
CBS 2499	12.5	27.05 ± 0.32^a^	61.36 ± 3.91^b^	95.92 ± 2.12^c^
	25	25.58 ± 1.43^a^	34.50 ± 2.19^a^	89.46 ± 5.65^b^
	50	25.23 ± 1.24^a^	23.40 ± 1.38^a^	78.22 ± 2.54^b^
	100	26.59 ± 1.61^a^	21.74 ± 1.14^b^	25.61 ± 0.67^a^
LO2e2	12.5	20.83 ± 1.04^a^	67.22 ± 1.97^b^	100.00 ± 0.47^c^
	25	21.71 ± 0.77^a^	34.51 ± 3.62^b^	95.92 ± 0.61^c^
	50	22.82 ± 2.16^a^	21.93 ± 0.39^a^	76.47 ± 1.48^b^
	100	22.73 ± 0.56^a^	22.68 ± 0.21^a^	22.73 ± 0.51^a^
CBS 4601	12.5	36.05 ± 2.42^a^	76.44 ± 1.69^b^	100.00 ± 0.62^c^
	25	37.49 ± 0.74^a^	44.55 ± 2.40^a^	97.62 ± 7.76^b^
	50	37.32 ± 1.00^a^	38.52 ± 4.38^a^	79.87 ± 2.37^b^
	100	38.25 ± 0.82^a^	35.70 ± 5.87^a^	38.19 ± 1.91^a^

Resistance, expressed as percentage, was reported as the ratio between the growth of treated samples and that of the untreated control. Data are the means of triplicate experiments ± standard deviation. Superscript letters indicate significant differences (*p* < 0.05) for each strain and chitosan type among the different concentrations of chitosan.

At the maximum allowed dose (100 mg/L), LO417 and CBS 4481 strains showed higher level of resistance than the other strains (between 54 and 67%) to all the chitosan types. Particularly, CBS 4481 strain was the most resistant one to all the chitosan types tested in this study, as indicated by the heat map, with patch color ranging from intense to light blue (Figure 2). These results demonstrate substantial strain-level variability in resistance to this antimicrobial. Consistent with earlier findings (Figure 1), shrimp shell chitosan exhibited the strongest antimicrobial activity across all tested strains and concentrations, significantly inhibiting cell growth compared to the controls. Furthermore, the resistance level was not affected by concentration of shrimp shells chitosan; in fact, no statistically significant differences were found in resistance levels among the different doses for any of the strains.

Conversely, the fungal chitosan, which is the product approved by OIV for oenological use, showed the lowest antimicrobial activity among the three types of chitosan; in fact, all the strains showed the highest percentage of resistance to this chitosan. For CBS 2499, LO2e2, and CBS 4601 strains, resistance levels were near or above 90% at lower concentrations (12.5 mg/L and 25 mg/L), indicated by blue patches in heat map (Figure 2), while a high growth reduction was observed in presence of 100 mg/L of chitosan, indicated by red patches in Figure 2, with exception of CBS 4481 strain. This trend highlights the rapid loss of effectiveness for fungal chitosan as the concentration decreases.

Insect-derived chitosan displayed a similar pattern of effectiveness to shrimp shell chitosan, particularly for LO417 and CBS 4481 strains, which were the highest chitosan-resistant strains. These strains showed resistance level similar to shrimp shell chitosan across all concentrations. As regards CBS 2499, LO2e2, and CBS 4601 strains, the resistance level to insect-derived chitosan increases significantly at the lowest concentration (12.5 mg/L), suggesting a reduction in effectiveness at lower doses.

In general, shrimp shell chitosan demonstrated the highest antimicrobial activity across all strains and concentrations. However, its use in oenology is restricted. The fungal chitosan, the form allowed for oenological use, diminished its effectiveness more rapidly as concentrations decrease, making it less reliable for controlling *B. bruxellensis* at lower doses.

Finally, insect-derived chitosan provided a viable alternative to shrimp shell chitosan, especially for LO417 and CBS 4481 strains, for which it remained effective even at lower concentrations. For other strains, it showed a reduction in effectiveness at the lowest concentration but still performed better than fungal chitosan.

### 3.2 Optical and electron microscopy

**Figure 3:**
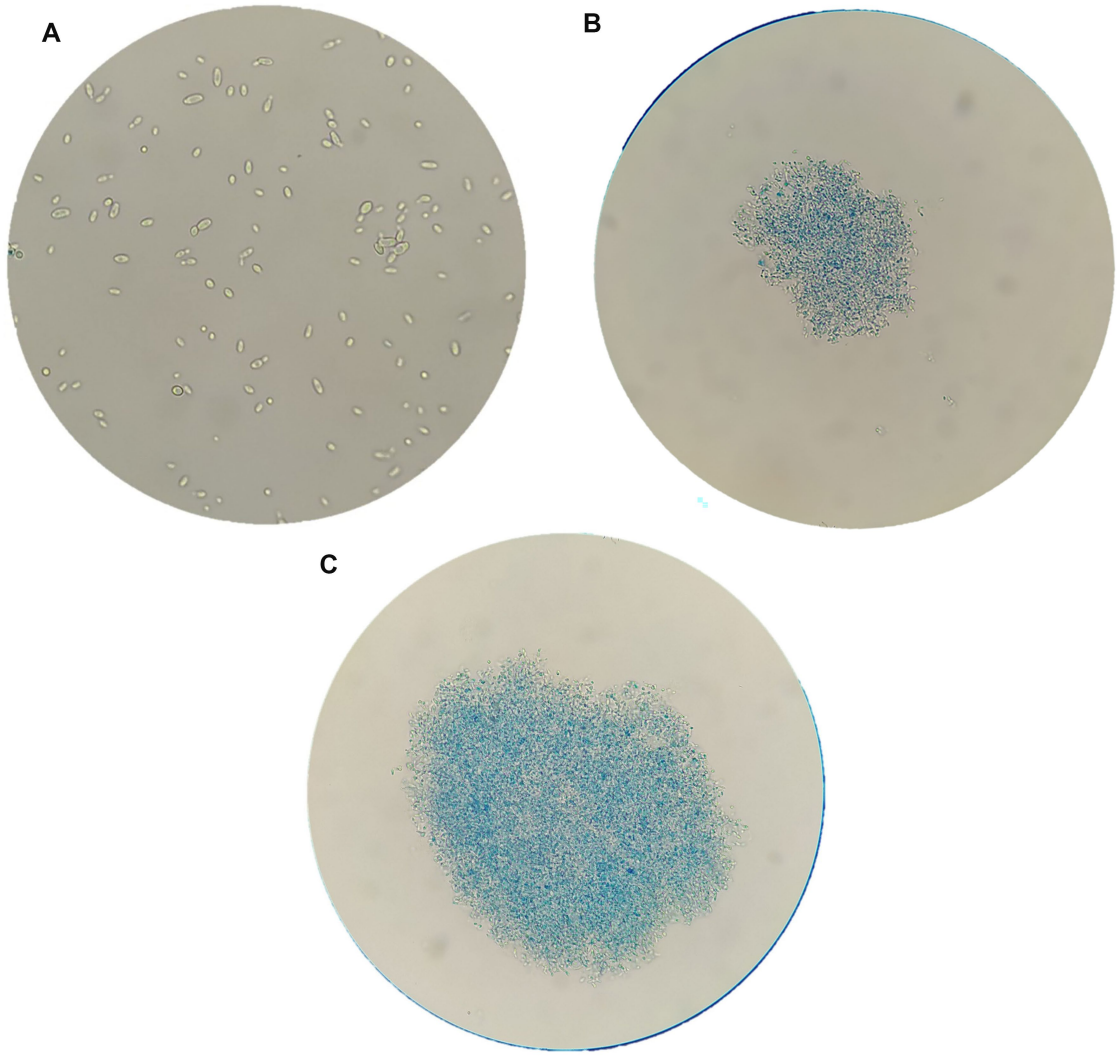
*Brettanomyces bruxellensis* (LO417) cells observed with optical microscope at 40X magnification, untreated **(A)** and treated with shrimp shells **(B)** and insect-derived chitosan **(C)**.

*Brettanomyces bruxellensis* strain cells, after chitosan treatments, were observed by optical and electron microscopy, in comparison to untreated cells. Figure 3 reports images of LO417 strain observed with optical microscope at 40X magnification after staining with methylene blue in different conditions, which were untreated cells (Figure 3A), treated with shrimp shells (Figure 3B) and insect-derived chitosan (Figure 3C). The blue colored cells were marked with methylene blue and were considered as dead; similar results were observed by Taillandier et al. (2015).

**Figure 4:**
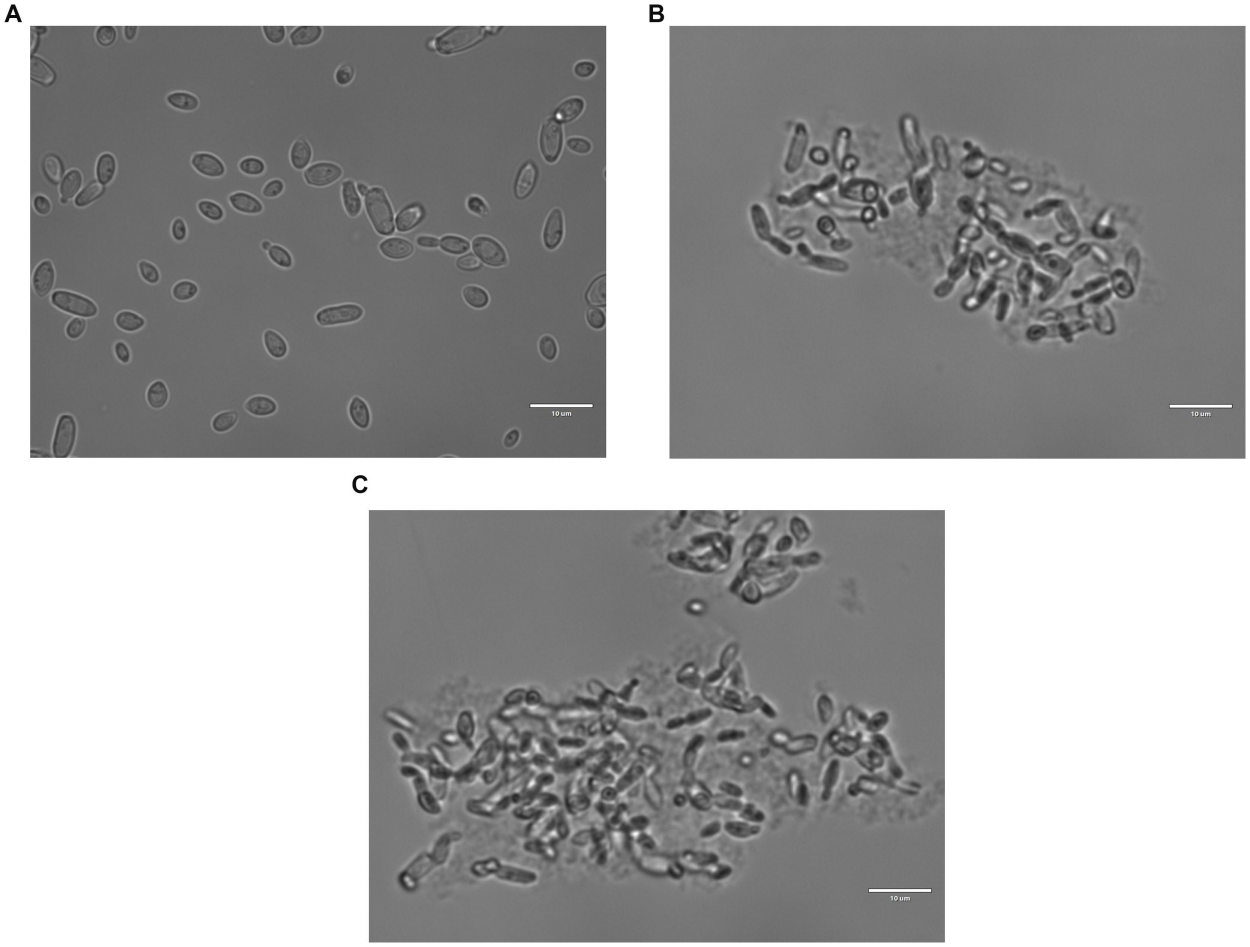
*Brettanomyces bruxellensis* (LO417) cells observed with optical microscope at 100X magnification, untreated **(A)** and treated with shrimp shells **(B)** and insect-derived chitosan **(C)**.

Figures 4A–C report images of the same strain in the same conditions, which were untreated (Figure 4A), treated with shrimp shells (Figure 4B) and insect-derived chitosan (Figure 4C), observed with 100X magnification. For both chitosan treatments, cells appeared aggregated due to the antimicrobial compound, whereas in the control, cells remained dispersed. This observation supports the hypothesis that chitosan’s antimicrobial action may result from electrostatic interactions between its positive charges and the negatively charged microbial cell surfaces, as previously reported by Verlee et al. (2017).

**Figure 5:**
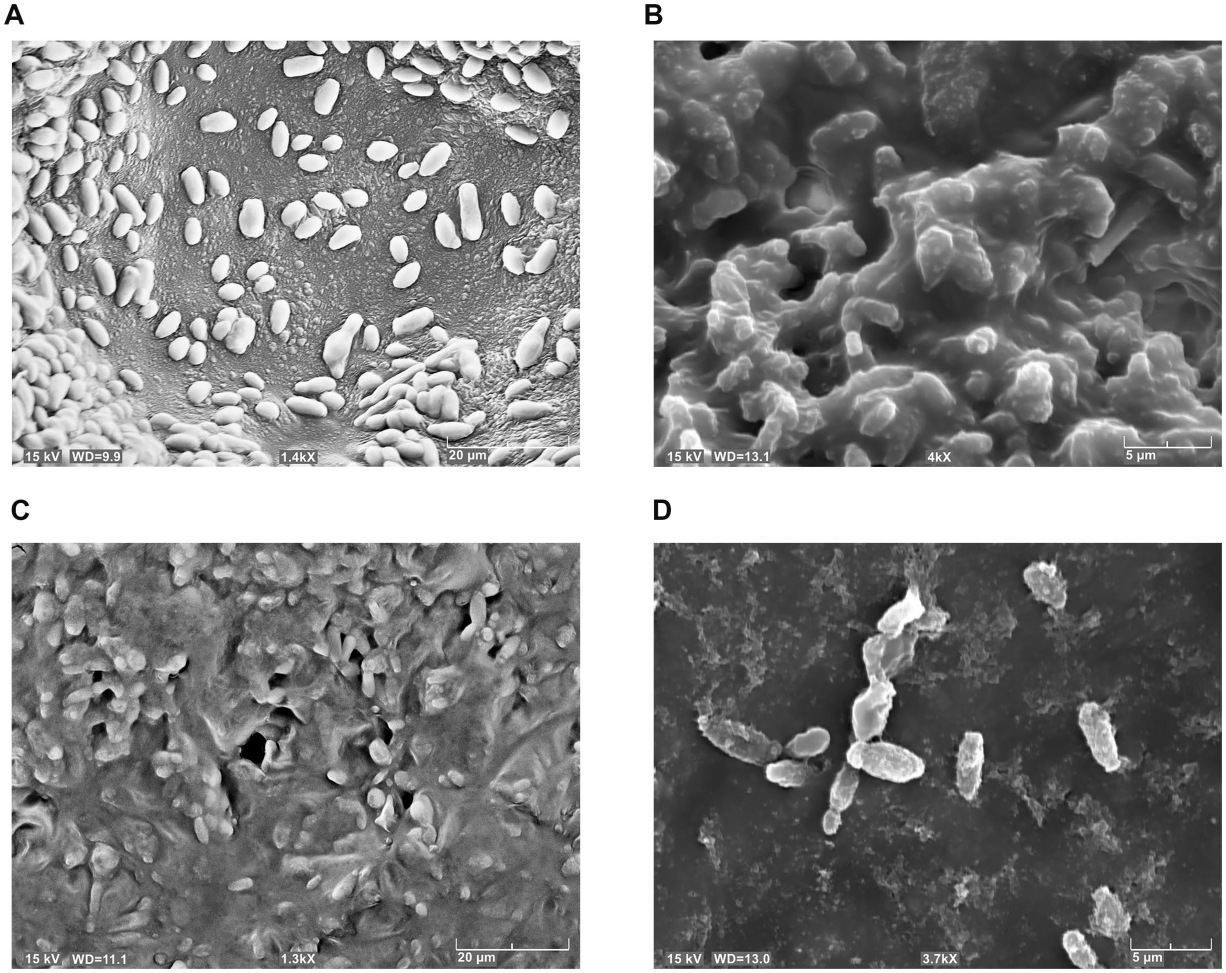
*Brettanomyces bruxellensis* (LO417) cells detected with scanning electron microscope, untreated **(A)** and treated with shrimp shells **(B)** and insect-derived chitosan **(C,D)**.

The phenomenon of cell aggregation was also observed by using the scanning electron microscope. Figure 5 showed the behavior of LO417 strain untreated (Figure 5A) and treated with the two chitosan types (Figures 5B–D). In addition to cell aggregation, deformation of surface could be observed on the treated cells (Figure 5D), with formation of small nodules on the surface treated with chitosan.

### 3.3 Chitosan activity against *Brettanomyces bruxellensis* and *Saccharomyces cerevisiae*

**Figure 6:**
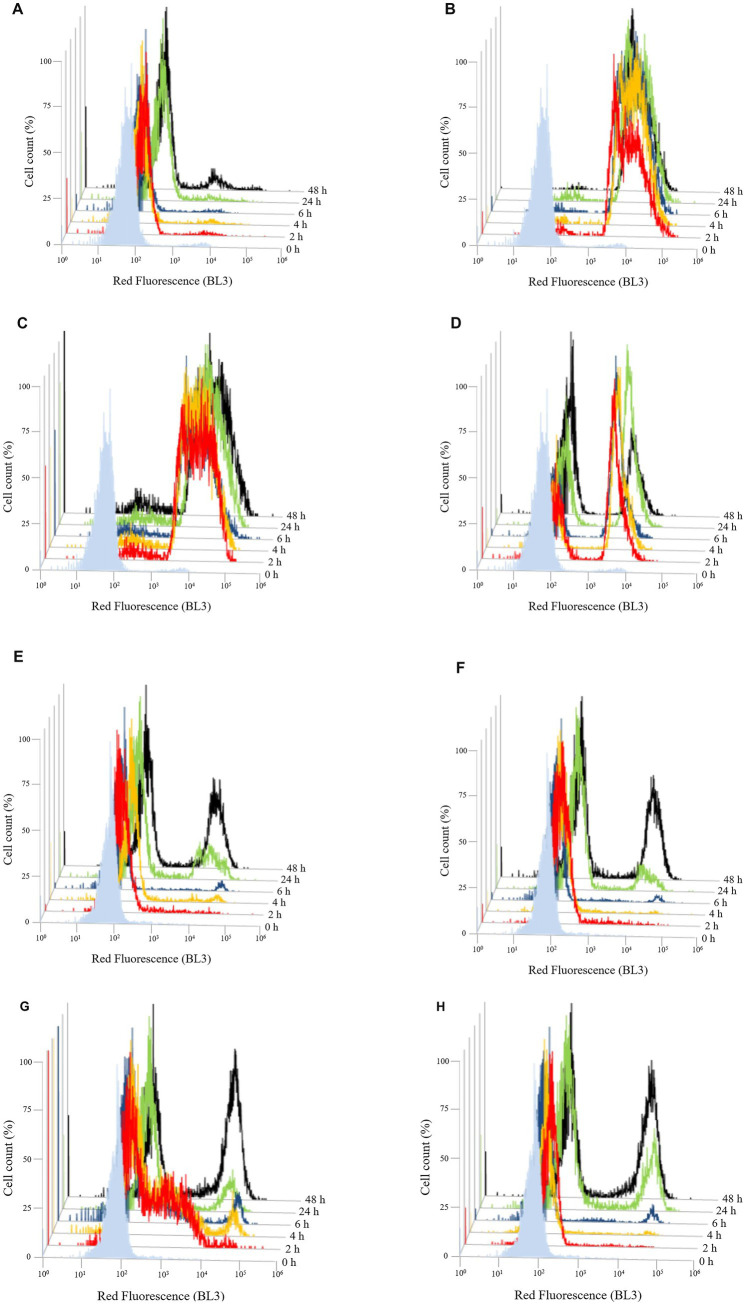
Changing of membrane permeability over the time of *Brettanomyces bruxellensis* LO417 **(A–D)** and *Saccharomyces cerevisiae* 4LBI-3 **(E–H)**. Membrane permeability was monitored by marking cells with propidium iodide. The following conditions were tested: untreated cells **(A,E)**; cells treated with chitosan from shrimp shells **(B,F)**; cells treated with insect-derived chitosan **(C,G)**; cells treated with fungal chitosan **(D,H)**.

**Figure 7:**
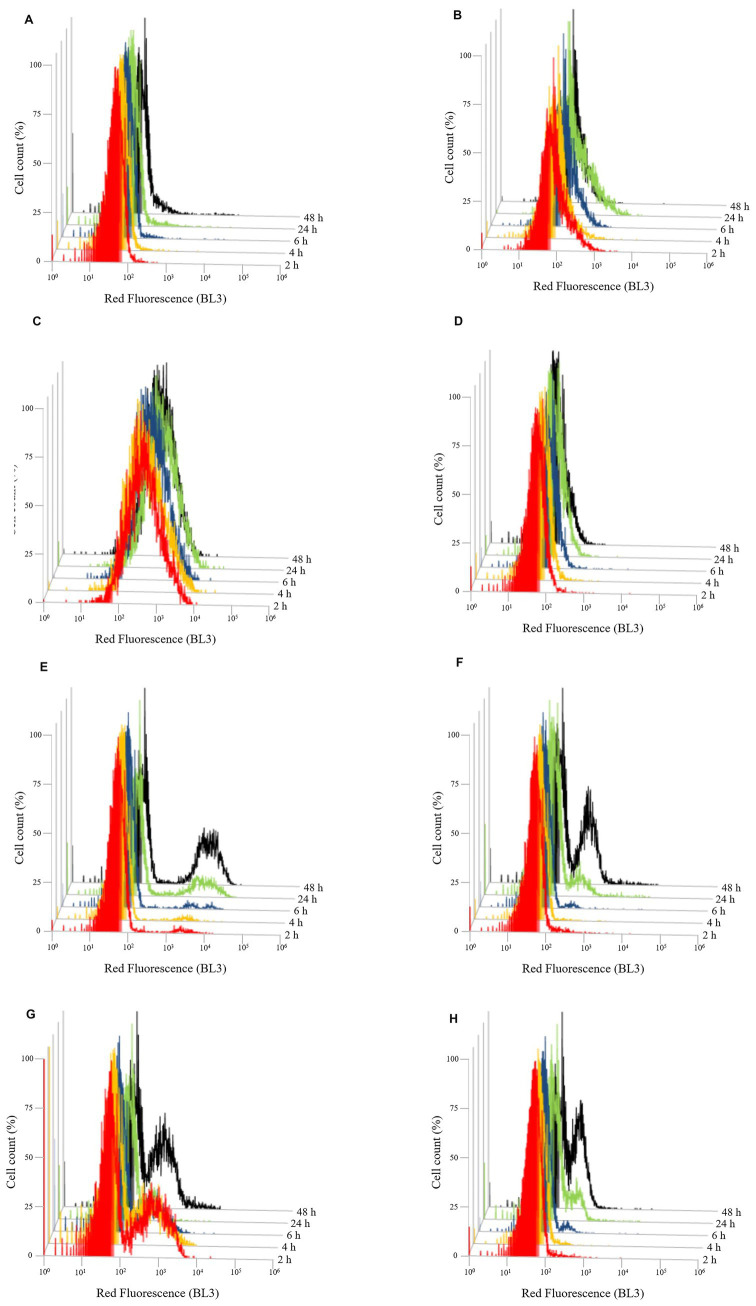
Evolution of cell death over the time of *Brettanomyces bruxellensis* LO417 **(A–D)** and *Saccharomyces cerevisiae* 4LBI-3 **(E–H)**. Cell death was monitored by marking cells with 7-AAD. The following conditions were tested: untreated cells **(A,E)**; cells treated with chitosan from shrimp shells **(B,F)**; cells treated with insect-derived chitosan **(C,G)**; cells treated with fungal chitosan **(D,H)**.

**Figure 8:**
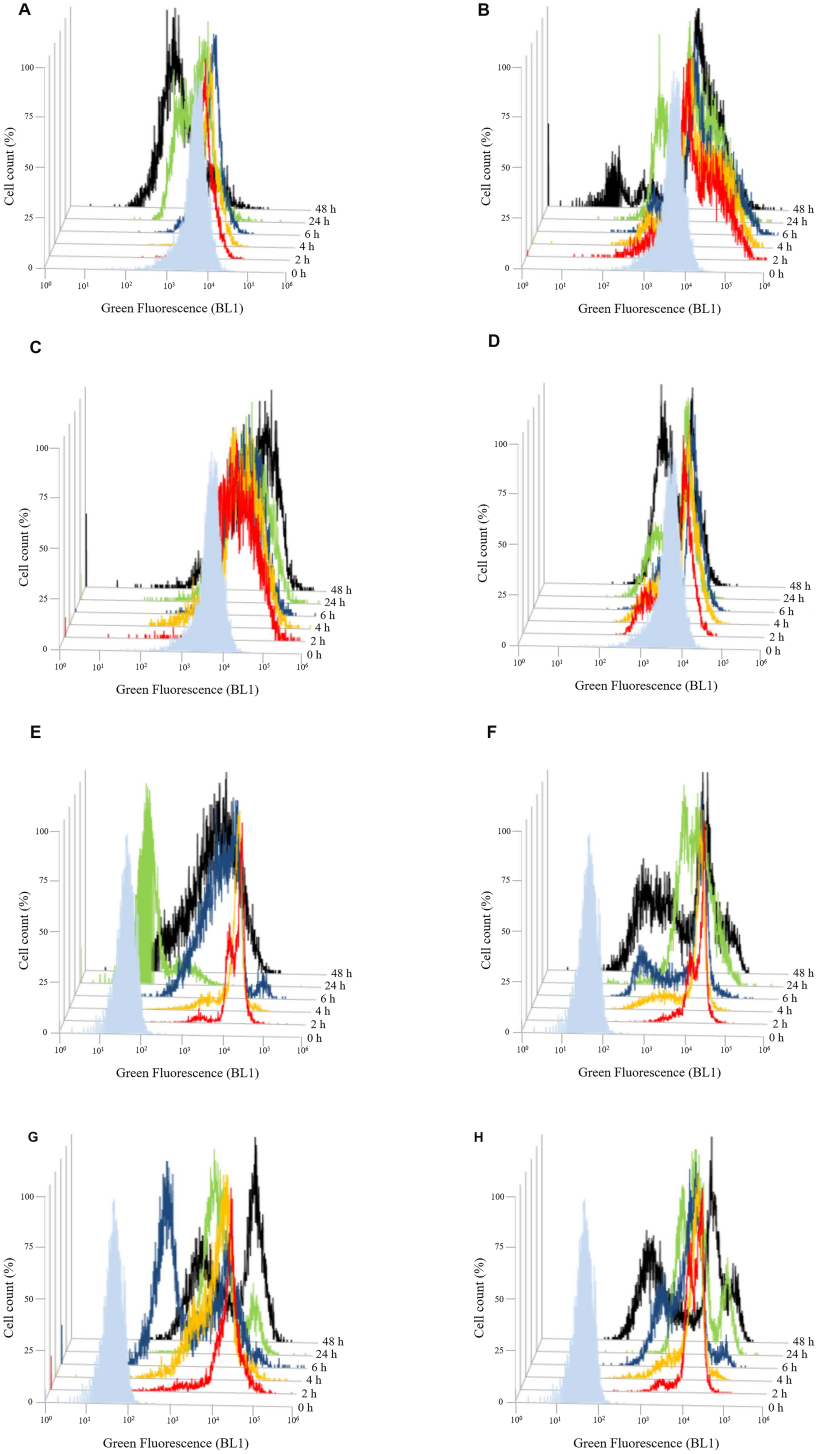
Evolution of membrane potential over the time of *Brettanomyces bruxellensis* LO417 **(A–D)** and *Saccharomyces cerevisiae* 4LBI-3 **(E–H)**. Membrane potential was monitored by marking cells with DiOC6(3). The following conditions were tested: untreated cells **(A,E)**; cells treated with chitosan from shrimp shells **(B,F)**; cells treated with insect-based derived chitosan **(C,G)**; cells treated with fungal chitosan **(D,H)**.

To evaluate the effect of the three different chitosan types (shrimp shell, insect-derived and fungal chitosan) on yeast cells, experiments were conducted on *B. bruxellensis* LO417 strain and *S. cerevisiae* 4LBI-3 strain. Membrane permeability, cell death and membrane potential were assessed over time using flow cytometry, with propidium iodide (PI), 7-Aminoactinomycin D (7-AAD) and DiOC_6_, respectively. PI results are shown in Figure 6 while 7-AAD results in Figure 7 and DiOC_6_ in Figure 8, with panels A–D referring to *B. bruxellensis* and E–H to *S. cerevisiae*.

Membrane integrity was first assessed by monitoring PI-positive cells (Figure 6). The control sample showed stable fluorescence in the first 48 h, indicating intact membranes. *B. bruxellensis* appeared more resistant than *S. cerevisiae* which showed membrane damage in 22.5% and 35.7% of cells at 24 and 48 h, respectively (Figures 6A,E). Treatment with shrimp shell (Figure 6B) and insect-based chitosan (Figure 6C) induced a rapid and significant increase in PI fluorescence in *B. bruxellensis*, reaching nearly 100% damaged cells within 2 h. Fungal chitosan (Figure 6D) produced a heterogeneous response, with both damaged and undamaged cells. A reduction in the damaged population at 48 h suggests partial recovery by a subpopulation of cells. In *S. cerevisiae*, cells treated with shrimp shells (Figure 6F) and fungal chitosan (Figure 6H), exhibited a characteristic double peak over the time, similar to the control, suggesting that both chitosan types did not significantly impact membrane permeability during the different growth phases. In contrast, cells treated with insect-based chitosan (Figure 6G) showed a marked shift in fluorescence intensity at 2 h, suggesting higher initial membrane damage. However, this effect appeared transient, as the overall trend aligned with the control over time. These findings suggest that shrimp shell and insect-based chitosan samples induce rapid and severe membrane damage in *B. bruxellensis*, whereas fungal chitosan elicits a more variable response. In *S. cerevisiae*, only insect-derived chitosan caused transient membrane disruption, with recovery over time.

Cell death, assessed by 7-AAD staining (Figure 7), supported membrane damage results. In *B. bruxellensis*, both the control (Figure 7A) and fungal chitosan-treated cells (Figure 7D) maintained low mortality levels. Insect-based chitosan (Figure 7C), however, led to a higher proportion of dead cells, while shrimp shell chitosan (Figure 7B) resulted in limited cell death despite strong membrane damage, indicating potential sublethal effects. In *S. cerevisiae,* all samples showed a slight increase in cell death over time, likely due to natural aging. Notably, only the insect-based chitosan treatment (Figure 7G) showed a distinct subpopulation of dead cells at 2 h, which decreased over time, suggesting partial recovery of cell viability.

Flow cytometry analysis using DiOC₆ (3) was performed to assess changes in membrane potential over time (Figure 8).

In *B. bruxellensis*, chitosan exposure resulted in a marked increase in fluorescence intensity with both shrimp shell (Figure 8B) and insect-based chitosan (Figure 8C) compared to the untreated control (Figure 8A). For shrimp shell chitosan, fluorescence levels decreased over time, suggesting a transient effect on membrane potential. Conversely, insect-based chitosan maintained high fluorescence throughout the first 24 h, indicating prolonged membrane perturbation. Fungal-derived chitosan (Figure 8D) induced a response comparable to the control, with no significant changes in fluorescence, suggesting minimal impact on membrane potential. In *S. cerevisiae*, the untreated control (Figure 8E) exhibited lower baseline fluorescence than *B. bruxellensis* strain (Figure 8A), reflecting intrinsic differences in membrane properties between the species. All chitosan-treated samples (Figures 8F–H) showed a gradual decrease in fluorescence intensity over the first 24 h, consistent with the trend observed in the untreated control (Figure 8E). This pattern suggests stable membrane potential and supports the overall viability of *S. cerevisiae* cell under all tested conditions.

### 3.4 Study of cell wall characteristics

To investigate whether the different responses of the two yeast species (*B. bruxellensis* and *S. cerevisiae*) could be related to variations in cell wall composition, specific cell wall characteristics were analyzed. In particular, surface charge and *β*-glucan content were measured for both strains.

The negative surface charge was evaluated by performing the percentage of Alcian blue retention (ABR %) test (Fukudome et al., 2002). The negative surface charge was significantly higher for *B. bruxellensis* LO417 strain than *S. cerevisiae* 4LBI-3 (Table 2).

**Table 2:** Percentage of Alcian blue retention (ABR %) and *β*-glucan content of *Brettanomyces bruxellensis* and *Saccharomyces cerevisiae* strains.

Yeast strain	Code	ABR %	β-glucan (g/100 g)
*B. bruxellensis*	LO417	55.01 ± 2.18^a^	9.50 ± 0.43^A^
*S. cerevisiae*	4LBI-3	7.17 ± 0.12^b^	3.26 ± 0.03^B^

Data are the means of duplicate experiments ± standard deviation. Superscript letters indicate significant differences (*p* < 0.05) among various strains. Lower case letters indicate differences in ABR % and capital letters indicate differences in β-glucan content.

The same result was observed for the β-glucan content, with a value much higher for *B. bruxellensis* than *S. cerevisiae*.

## 4 Discussion

This work explored the effect of chitosan from different sources on some *Brettanomyces bruxellensis* strains. Other than the commercial formulation of chitosan approved for oenological use, having fungal origin, chitosan from shrimp shells (a chemical defined product, commercially available) and insects (an innovative source) were evaluated. The effectiveness of the tested chitosan types in reducing *Brettanomyces* population appears to depend both on strain sensitivity and chitosan source. The highest antimicrobial activity was exhibited by chitosan derived from shrimp shells, which exhibits a strong antimicrobial effect across all the concentrations for all *B. bruxellensis* strains. However, the use of this chitosan type in oenology is restricted in consequence of the potential presence of some proteins, i.e., tropomyosin, responsible for intoxication problems in sensitive individuals (Amaral et al., 2016). This issue has been solved by using chitosan from sources other than crustaceans, such as chitosan from zygomycetes, which in our study was effective but only at doses higher than 50 mg/L.

The new alternative source of chitosan tested in this research, the insect-derived chitosan, was more effective in reduction of *Brettanomyces* population than fungal chitosan, with high efficacy also at low concentrations. Recently, it was reported that one of the advantages of insect-derived chitosan lies in its potential to reduce allergenicity compared to chitosan derived from crustaceans. The presence of allergenic proteins in chitosan from crustacean sources can be responsible for the allergic reactions to chitosan from these sources. The different protein composition of insects might reduce the risk of allergic reactions in crustaceans-sensitive individuals (Mishyna and Glumac, 2021).

Although the highest activity was observed for shrimp shell chitosan, a similar pattern of effectiveness was observed for insect-derived chitosan. These results confirm literature data reporting that insect-derived chitin/chitosan generally have properties similar to those obtained from crustaceans (Philibert et al., 2017).

In addition to the source, the antimicrobial activity of chitosan is dependent on characteristics of the biopolymer, such as molecular weight (MW) and the deacetylation degree (DA) as the lower molecular weight chitosan with a higher deacetylation degree was shown to be more efficient (Miot-Sertier et al., 2022).

Generally, it was reported that chitosan with a higher deacetylation degree, and consequently higher positive charge, is more soluble in acidic media and would be expected to have a stronger antimicrobial activity than lower deacetylation degree chitosan (Takahashi et al., 2008; Hosseinnejad and Jafari, 2016; Bertrand et al., 2024). Our results confirm these findings. Indeed, the lowest activity of fungal chitosan might be related to the lower deacetylation degree of this formulation (>60%) in comparison to shrimp shell and insect-derived (>75% and >90%, respectively) chitosan types.

As regards the molecular weight (Mw), usually it ranges between 20 kDa and 2,000 kDa and chitosans can be classified as low Mw (<100 kDa), medium Mw (100–1,000 kDa) and high Mw (1,000–2,000 kDa) and this classification influences greatly some of chitosan properties (Petroni et al., 2023; Román-Doval et al., 2023). Chitosan with medium-low Mw has generally been reported to exhibit stronger antibacterial activity than high Mw forms, likely due to its greater ability to penetrate bacterial cell walls and interfere with metabolic processes (Zivanovic et al., 2004; Vishu Kumar et al., 2005). In contrast, some studies suggest that high Mw chitosan may form an external barrier that impedes nutrient uptake by bacteria, provoking bacterial death (Zheng and Zhu, 2003; Hosseinnejad and Jafari, 2016). Furthermore, it is reported that the effect on Gram-negative bacteria is different from Gram-positive response. As regards the antifungal activity, it was reported that the influence of chitosan characteristics was dependent on fungus type (Ziani et al., 2009). A decrease in fungal growth with increasing Mw was reported for *Fusarium oxysporum*, while for *Aspergillus niger* no influence of Mw was observed.

In our case, the highest efficacy of insect-based chitosan might be related to its lowest molecular weight, in comparison to the two other formulations.

Other factors affecting the effectiveness of chitosan tested in our study were the dose and strain sensitivity. Except for shrimp shells chitosan, the effect of chitosan was concentration dependent, although the inhibiting level was variable between fungal and insect-derived chitosan. Fungal formulation was effective at the highest concentration tested in this study (100 mg/L, Figure 1) for all the strain with exception of LO147 strain, while the effective concentration for insect-derived chitosan was variable among the different strains. However, the chitosan sensitivity appears to be an intrinsic characteristic of each strain, with CBS 4481 and LO417 (to a lesser extent) resulting in the highest chitosan-resistant strains. It was previously reported that the sensitivity of a given strain appears to be a specific characteristic of the strain, and it is poorly affected by other factors, such as strain physiological state or fungal chitosan dose (Ferreira et al., 2013; Paulin et al., 2020). The variable sensitivity/resistance of a yeast might be related to the presence of surface elements or specific cell wall component which allow the entrance/binding of chitosan in the cell or compounds protecting microbial cells from chitosan (Palma-Guerrero et al., 2010; Jaime et al., 2012).

The microscopic observation of yeast cells treated with chitosan in comparison to the control sample, coupled with methylene blue coloration, confirms the involvement of the yeast cell wall in chitosan mechanism of action. In fact, treated cells were aggregated by the antimicrobial compound, clearly showing adsorption phenomenon, whereas untreated cells were separated from each other (Figure 3). These results agree with several studies reporting that the antimicrobial action of chitosan may imply a direct contact between the yeast’s cell wall and the polysaccharide (Goy et al., 2009; Kong et al., 2010; Taillandier et al., 2015; Verlee et al., 2017).

The electron microscopy observation of cells, other to confirm adsorption phenomena between chitosan and yeast cells (Figure 5), revealed a changing in surface of treated cells. In detail, cells treated with chitosan developed small “bumps” not present in untreated cells. Similar results were already reported by Petrova et al. (2016) for *B. bruxellensis* strains, while Park et al. (2008) reported that *Candida albicans* and *Fusarium oxysporum* treated with chitosan showed cell surface disruption, while a normal smooth surface was observed for untreated cells.

In order to better investigate the effect of the chitosan on yeast cells, two indigenous strains, one belonging to *B. bruxellensis* (LO417), the main microbial target of chitosan treatment in wine, and one belonging to *S. cerevisiae* (4LBI-3), a species considered as chitosan resistant (Roller and Covill, 1999; Bağder Elmacı et al., 2015) were analyzed by flow cytometry. The monitoring of membrane permeability over time by detecting the cells marked with PI (Figure 6) confirmed the effect of chitosan on membrane permeability for the chitosan sensitive species, while no membrane damage was observed for the chitosan resistant species. Indeed, it was demonstrated (Li et al., 2015) that one of the antimicrobial activities of chitosan was due to the interactions of cationic NH_3_^+^ groups with negatively charged cell membranes, with consequent increase of membrane permeability and membrane lysis. However, a different activity was observed in function of chitosan source, with significant membrane damage in LO417 strain by shrimp shells and insect-derived chitosan, while a high percentage of cell death was observed for insect-derived chitosan. Shrimp shells chitosan caused membrane permeabilization without a corresponding high level of cell death, suggesting a different mechanism of action or cell response. Fungal chitosan, despite causing some membrane damage, did not lead to substantial cell death, indicating its limited effectiveness compared to the other types. Other authors (Jeihanipour et al., 2007) reported a lower antimicrobial activity of fungal chitosan compared to chitosan from crustaceans. The effect on yeast cell by insect-derived chitosan might be due to its low molecular weight; it was widely demonstrated that the diffusion of low molecular weight chitosan into the cell and its interaction with DNA, RNA, and proteins contribute to cell damage (Liu et al., 2004; Zakrzewska et al., 2005; Eaton et al., 2008; Li et al., 2010; Taillandier et al., 2015).

As regards the effect on *S. cerevisiae*, it appeared that the insect-derived chitosan had affected the initial population (Figures 6G, 7G). However, it can be assumed that, as reported by Guzzon et al. (2022), the high growth rate of *S. cerevisiae* could lead to the development of a viable population from the few cells that survived after contact with chitosan, thus observing a trend similar to the control.

It is reported that the antifungal properties of chitosan are mainly related to the interaction of chitosan with the cell wall or cell membrane. Different studies report the correlation between unsaturated fatty acid contents of cell membrane and chitosan resistance (Palma-Guerrero et al., 2010); indeed, the chitosan susceptibility is positively correlated with content of unsaturated fatty acid as a higher content of unsaturated fatty acids increases the membrane fluidity, leading to a more negative charge on the cell membrane (Kumariya et al., 2015). In our study, the effect of other characteristics of cell walls were investigated, which were the differences in the surface charge and *β*-glucan content, on chitosan resistance of the two analyzed yeast species. High differences in cell wall characteristics were detected between the two species, confirming that the cell walls of different microorganisms, species or even strains vary considerably in their overall composition, which leads to varying adsorption capacity. Other authors reported that the *β*-glucan levels in cell wall were strongly dependent on yeast species (Kang et al., 2014). The higher negative surface charge of *B. bruxellensis* than *S. cerevisiae* might explain the lower chitosan resistance of *Brettanomyces*. Indeed, it was reported that the interactions between positively charged molecules of chitosan and negatively charged molecules of microbial cell walls affects the cell membrane structure and permeability, inducing leakage of intracellular materials, with modification of biochemical and physiological activity of microbial cell, with consequent loss of growth capacity and death (Shahidi et al., 1999).

## 5 Conclusion

The results of this study confirmed the antimicrobial activity of chitosan against *Brettanomyces bruxellensis* strains, but it was demonstrated the high influence of chitosan source on the efficacy of this compound. The use of different experimental procedures, such as biochemical assays and flow cytometry, revealed the higher antimicrobial activity of chitosan extracted from shrimp shells and insect-based chitosan in comparison to the chitosan of fungal origin, currently approved for oenological use. Furthermore, the flow cytometry analysis indicated a different mechanism of action or cell response in function of the chitosan source. Indeed, all the tested chitosan types determined a membrane damage in *Brettanomyces* cells, but high level of dead cells was observed only for insect-based chitosan, while fungal chitosan did not lead to substantial cell death, confirming the lowest effectiveness compared to the other types.

These findings underscored the potential of insect-based chitosan as an effective antimicrobial agent for *Brettanomyces* control in wine, offering an effective solution for protection of wine from *Brettanomyces* contamination. However, further work will be necessary in order to optimize its use during the different stages of winemaking and to evaluate its effects on wine sensorial properties, other than to ascertain all the aspects related to food safety.

## Data Availability

The original contributions presented in the study are included in the article/supplementary material, further inquiries can be directed to the corresponding author.
